# Transcriptional profiling of *Xanthomonas campestris* pv. c*ampestris* in viable but nonculturable state

**DOI:** 10.1186/s12864-023-09200-z

**Published:** 2023-03-09

**Authors:** Kaihong Bai, Xiaoli Xu, Xudong Wang, Yao Li, Chengxuan Yu, Na Jiang, Jianqiang Li, Laixin Luo

**Affiliations:** 1 School of Life Sciences, Zhengzhou University, Zhengzhou, Henan 450001 People’s Republic of China; 2 Department of Plant Pathology, Key Laboratory of Seed Disease Testing and Control, MOA Key Lab of Pest Monitoring and Green Management, China Agricultural University, No.2 Yuanmingyuan West Road, Haidian District, Beijing, 100193 People’s Republic of China

**Keywords:** *Xanthomonas campestris* pv. *campestris*, RNA-seq, VBNC state

## Abstract

**Background:**

*Xanthomonas campestris* pv. c*ampestris* (Xcc) is an important seed-borne plant pathogenic bacteria that can cause a serious threat to cruciferous crops. Bacteria can enter into the viable but non-culturable (VBNC) state under stress conditions, and cause potential risks to agricultural production because the VBNC bacterial cells will evade culture-based detection. However, little is known about the mechanism of VBNC. Our previous study showed that Xcc could be induced into VBNC state by copper ion (Cu^2+^).

**Results:**

Here, RNA-seq was performed to explore the mechanism of VBNC state. The results indicated that expression profiling was changed dramatically in the different VBNC stages (0 d, 1 d, 2 d and 10 d). Moreover, metabolism related pathways were enriched according to COG, GO and KEGG analysis of differentially expressed genes (DEGs). The DEGs associated with cell motility were down-regulated, whereas pathogenicity related genes were up-regulated. This study revealed that the high expression of genes related to stress response could trigger the active cells to VBNC state, while the genes involved in transcription and translation category, as well as transport and metabolism category, were ascribed to maintaining the VBNC state.

**Conclusion:**

This study summarized not only the related pathways that might trigger and maintain VBNC state, but also the expression profiling of genes in different survival state of bacteria under stress. It provided a new kind of gene expression profile and new ideas for studying VBNC state mechanism in *X. campestris* pv. *campestris*.

**Supplementary Information:**

The online version contains supplementary material available at 10.1186/s12864-023-09200-z.

## Background

*Xanthomonas campestris* pv. *campestris* (Xcc) is the pathogen of black rot of crucifers. It is an important seed-borne bacterium and causes significant losses in crucifers worldwide [1]. Xcc is a representative Gram-negative phytopathogenic bacterium and possesses a single polar flagellum. It also can produce xanthan gum and secrete cellulases, proteases and amylases [2].

The viable but nonculturable (VBNC) state of bacteria was first reported in *Escherichia coli* and *Vibrio cholerae* in 1982 [3]. The VBNC cells are viable, but could not be detected by conventional culture-based techniques. These cells can cause potential risks to agricultural production through evading culture-based detection. Unfavorable environmental stimuli could induce the VBNC state, e. g. low temperature, limited nutrition, non-optimal pH, low oxygen availability, and heavy metal exposure [4]. When the adverse conditions were removed, VBNC cells could be resuscitated and infect the host again. For VBNC cells, the cell wall structure, membrane fatty acids and genes expression related to stress response could change, macromolecular synthesis and respiration rate could decrease, and ATP levels and membrane potential could increase [4–6]. There were more than one hundred species of bacteria have been reported the VBNC state over the past 40 years, including many plant pathogens such as *Agrobacterium tumefaciens*, *Ralstonia solanacearum*, Xcc, *Erwinia amylovora*, *X. axonopodis* pv. *citri*, *Pseudomonas syringae* pv. *syringae* and *P. syringae* pv. *tabaci*, *Xylella fastidiosa*, *Clavibacter michiganensis*, and *Acidovorax citrulli* [7–15]. For Xcc, VBNC state could be induced by copper, sterile soils and oligotrophic conditions [9, 16, 17].

Although the VBNC state is generally regarded as a survival strategy to against adverse conditions [4], the molecular mechanism is less reported. In previous reports, the transcriptomic approach was used to explore the mechanism of VBNC state in *E. coli* [18–20], *Brettanomyces bruxellensis* [21], *V. alginolyticus* [22], *V. cholerae* [23], *Corynebacterium diphtheriae* [24], *P. syringae* pv. *syringae* [25], *Enterococcus faecalis* [26], *V. parahaemolyticus* [27], and *C. michiganensis* [28]. Here, RNA-seq was also performed to explore the mechanism of VBNC state in *Xanthomonas campestris* pv. *campestris* under copper induction.

## Results

### Assessment of RNA-Seq read quality

Total RNA samples were collected at various time points (0 d, 1 d, 2 d and 10 d) after induction of the VBNC by 50 µM CuSO_4_ and subjected to RNA-Seq analysis. The data produced consisted of a total of 317,177,358 raw reads (PRJNA797910, SRA, NCBI), which yielded 316,142,066 clean reads with 47,187,376,274 clean bases after low-quality reads had been removed by filtering to produce samples having Q20 values greater than 98.82% (Table 1).

**Table 1:** Summary of RNA-seq data

Sample time	Sample ID^a^	Raw reads	Clean Reads	Clean Bases (bp)	Q20 (%)^b^	Q30 (%)^c^
0 min	CK_1	31,068,304	30,962,674	4,623,897,717	98.92	96.44
	CK_2	35,955,456	35,842,100	5,355,651,336	98.92	96.44
	CK_3	23,758,872	23,681,260	3,535,242,993	98.97	96.58
1 d	C1_1	25,734,326	25,646,982	3,827,149,923	98.87	96.32
	C1_2	24,366,996	24,295,288	3,625,750,139	98.99	96.66
	C1_3	23,503,642	23,428,398	3,495,853,547	98.93	96.46
2 d	C2_1	25,391,976	25,313,122	3,778,073,309	98.87	96.26
	C2_2	26,882,156	26,789,250	3,999,859,294	98.88	96.33
	C2_3	25,579,618	25,498,868	3,806,817,290	98.89	96.34
10 d	C3_1	25,219,426	25,135,710	3,748,176,994	98.85	96.32
	C3_2	25,308,130	25,227,920	3,764,168,671	98.92	96.49
	C3_3	24,408,456	24,320,494	3,626,735,061	98.82	96.23
Total		317,177,358	316,142,066	47,187,376,274		

^a^ 1, 2 and 3 represent three independent biological replicates

^b^ Q20: The percentage of bases with a Phred value > 20

^c^ Q30: The percentage of bases with a Phred value > 30

### Overview of gene expression profiles

**Fig. 1:**
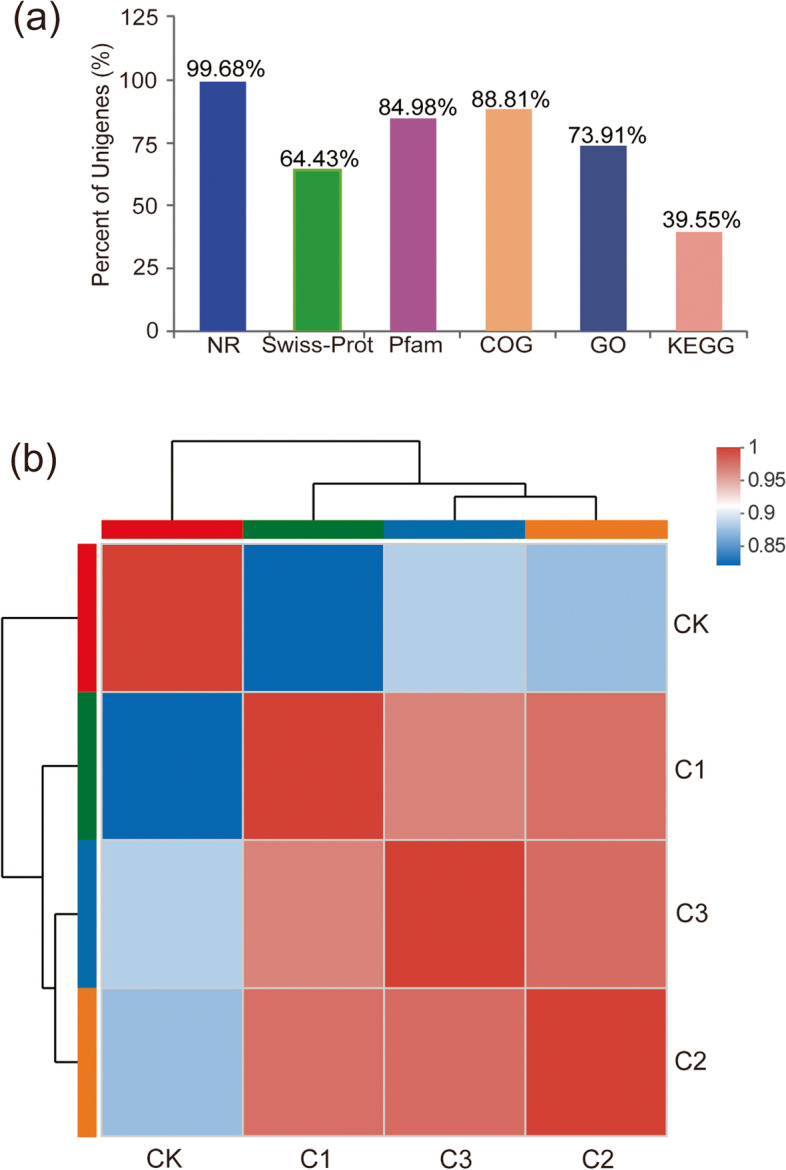
Overview of RNA-seq data. The top panel (**a**) depicts the percent of unigenes in the NR, Swiss-Prot, Pfam, COG, GO and KEGG databases. The heat map of average gene expression level (**b**) shows the similarity among CK, C1, C2 and C3. CK indicates time zero, while C1-C3 indicate three time points after exposure to copper each associated with a different stage in the establishment of the VBNC state, namely early (1 d) and developing (2 d) stage induction, and the stable VBNC state (10 d), respectively

The RNA-seq data indicated that the genome size was 5,148,708 bp, 4258 CDS were yielded, and GC content was 64.96%. The expressed genes were counted in various databases and there were 4258 genes (99.68%), 2756 genes (64.43%), 3631 genes (84.95%), 3795 genes (88.81%), 3158 genes (73.91%) and 1690 genes (39.55%) annotated in NR, Swiss-Prot, Pfam, COG, GO and KEGG databases, respectively (Fig. 1a). Previous data showed all culturable cells could be induced into VBNC state at 1 d [17]. Thus, the samples at 1 d and 2 d were considered to represent the early (C1) and developing (C2) stages of the VBNC state, respectively, and the sample at 10 d represented the stable VBNC state (C3), while CK indicated the log-phase cells at time zero. The heat map of average gene expression level showed that the expression patterns of C2 and C3 were most similar and C1 could gather together in a large clade with C2 and C3, whereas CK was significantly the most different with C1, C2 and C3, which meant that expression pattern was changed dramatically over time of copper induction (Fig. 1b).

### Functional annotation analysis of differentially expressed genes in the VBNC state

**Fig. 2:**
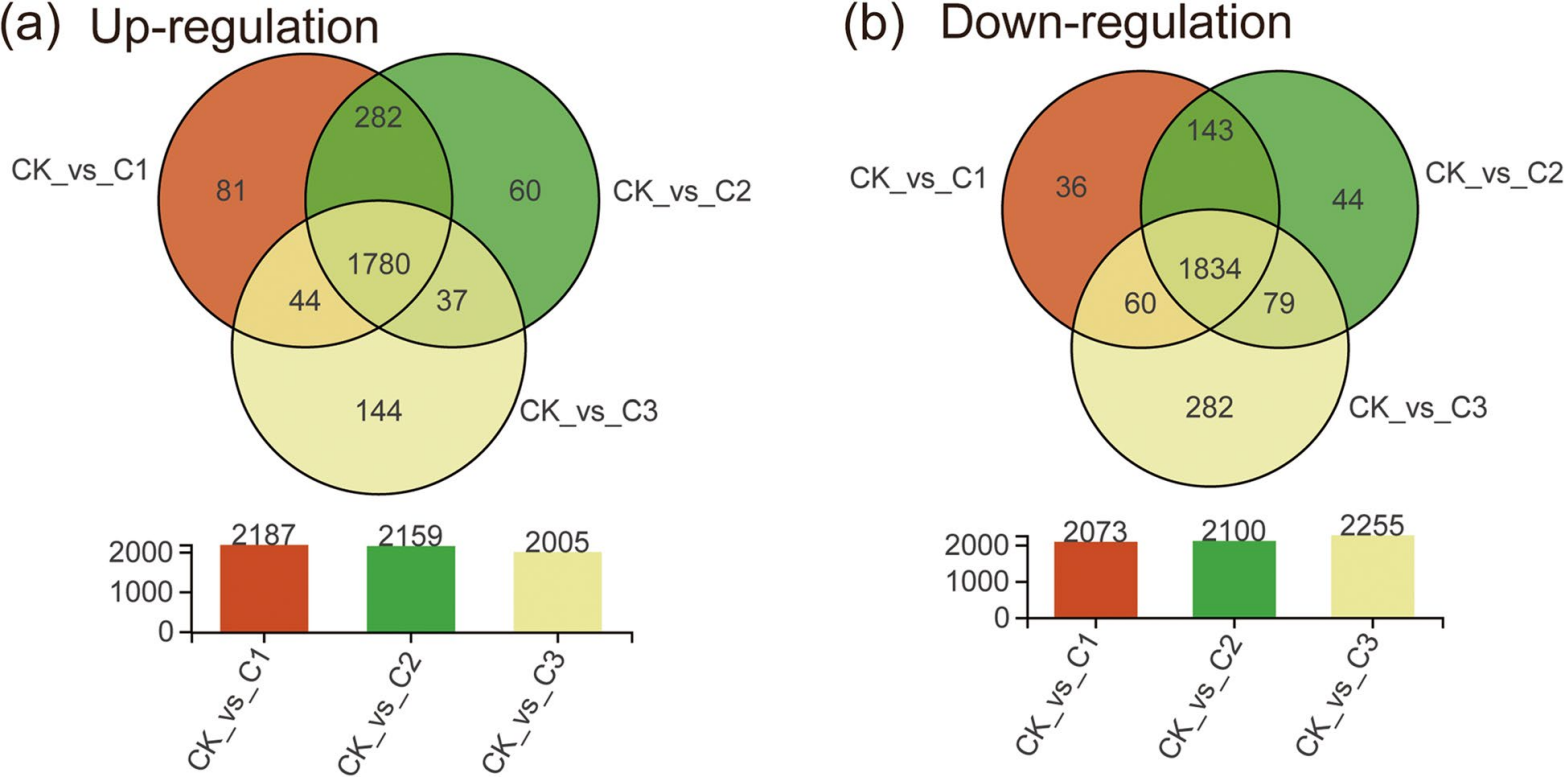
Gene expression profiles of differentially expressed genes from four different stages by copper exposure. The left panel (**a**) and right panel (**b**) shows the up-regulated genes and down-regulated genes, respectively. The above graph indicates the Venn diagram of differentially expressed genes in comparison to time zero for three different time points (1 d, 2 d, and 10 d) and the bottom bar chart shows the number of differentially expressed genes. CK indicates time zero, while C1-C3 indicate three time points after exposure to copper each associated with a different stage in the establishment of the VBNC state, namely early (1 d) and developing (2 d) stage induction, and the stable VBNC state (10 d), respectively

Differentially expressed genes (DEGs) were identified in 1 d (C1), 2 d (C2), and 10 d (C3) in comparison of the cells in the log-phase at time zero (CK). The results of gene expression fold changes (FC) values revealed that 1780 genes were significantly up-regulated at all three tested time points after exposure to copper, and 1834 genes were significantly down-regulated (Fig. 2).

**Fig. 3:**
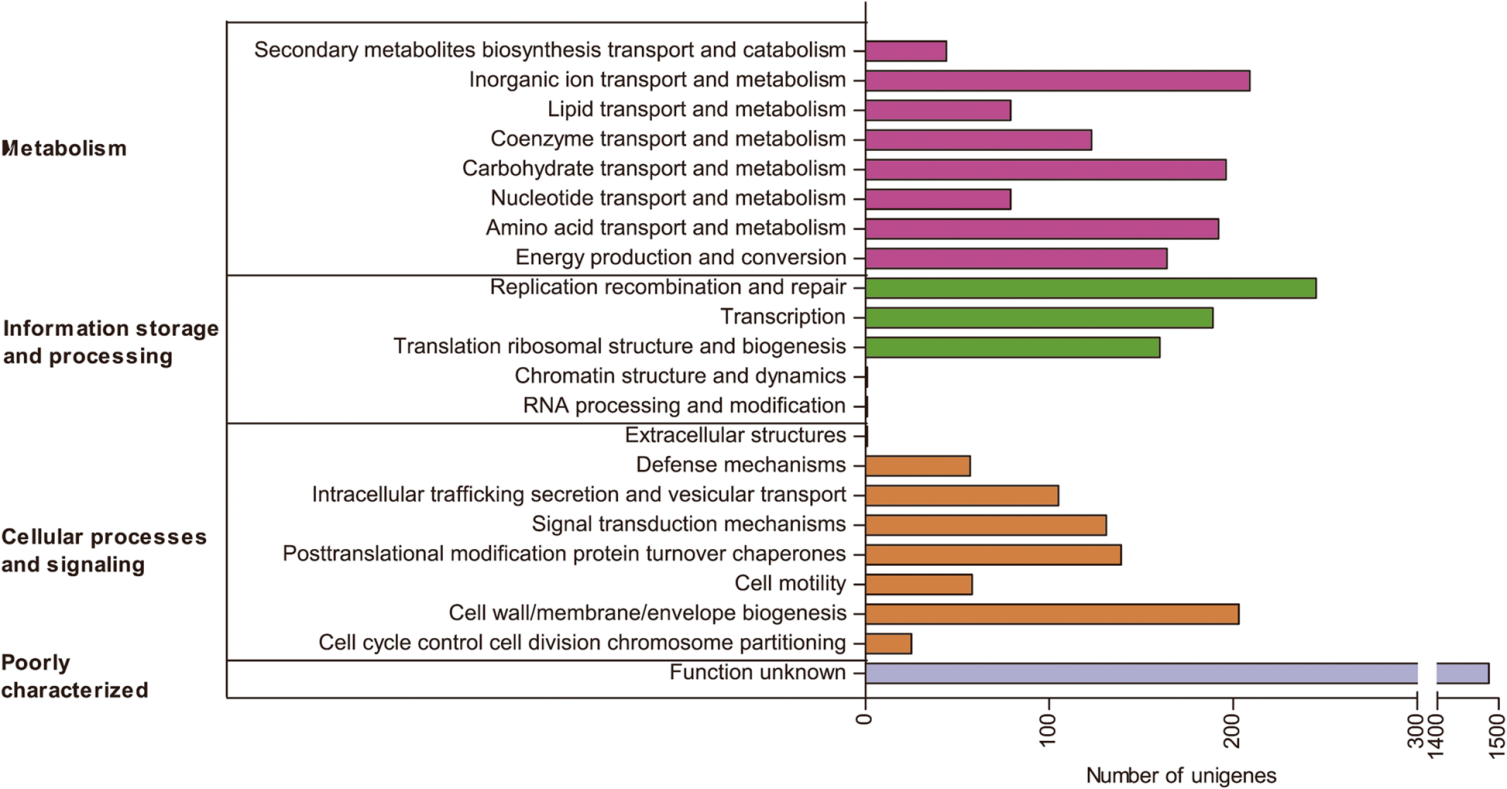
COG enrichment of differentially expressed genes among four different stages by copper exposure. Four different stages indicate 0 d, 1 d, 2 d and 10 d after exposure to copper

**Fig. 4:**
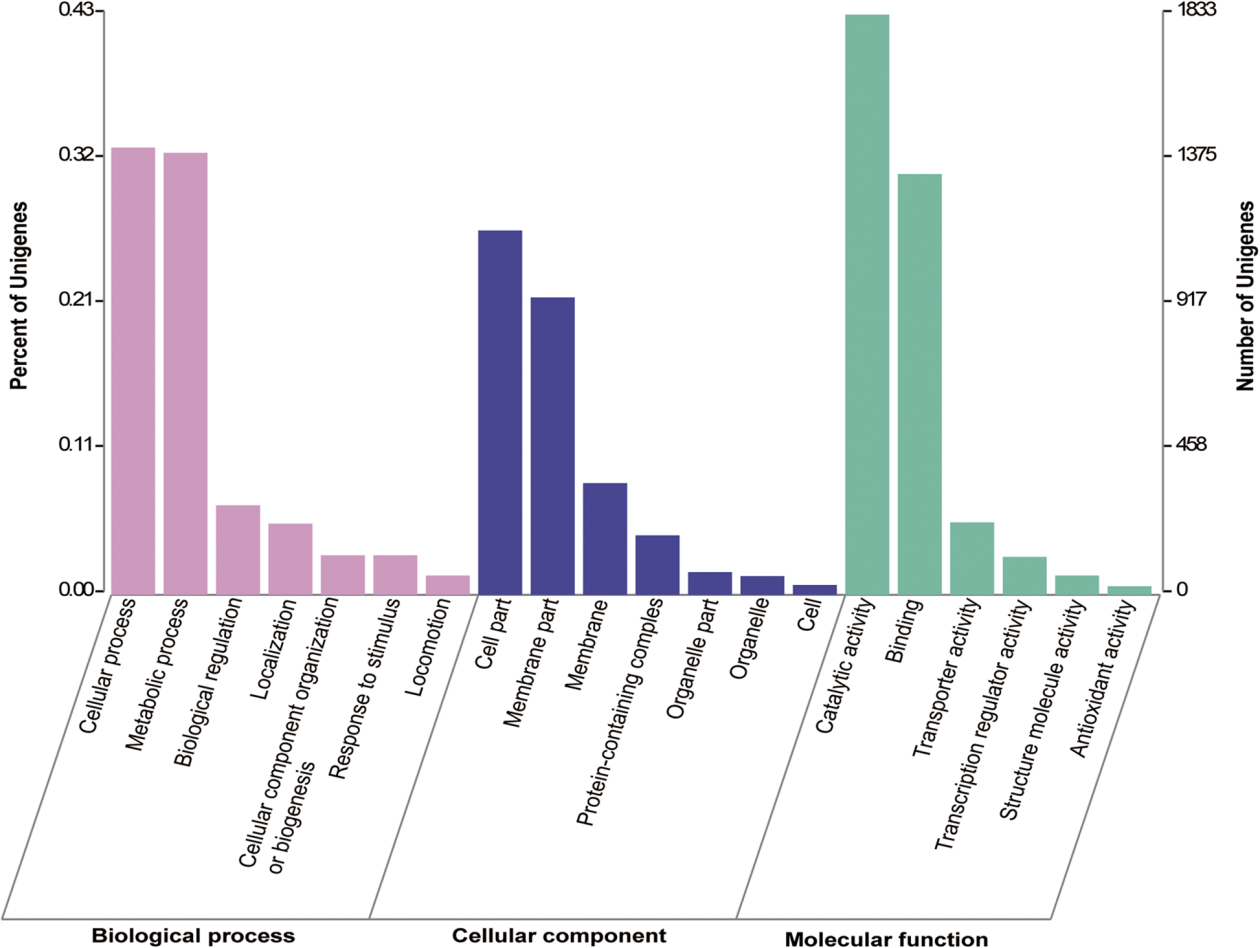
GO enrichment of differentially expressed genes among four different stages by copper exposure. Four different stages indicate 0 d, 1 d, 2 d and 10 d after exposure to copper

**Fig. 5:**
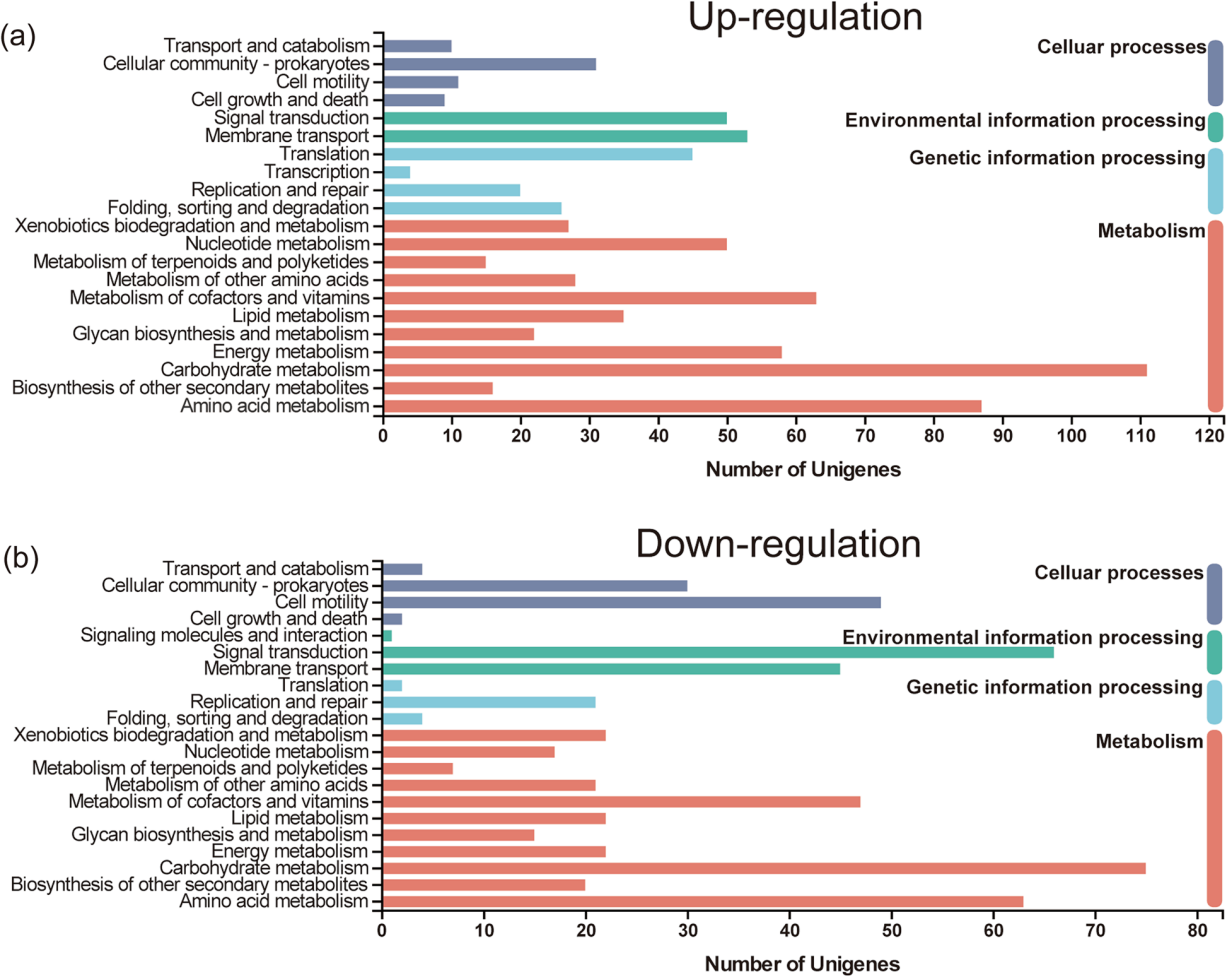
KEGG pathway enrichment analysis of the differentially expressed genes among four different stages by copper exposure. Four different stages indicate 0 d, 1 d, 2 d and 10 d after exposure to copper. The above panel (**a**) and bottom panel (**b**) indicate the up-regulated expression genes and down-regulated expression genes, respectively

The DEGs were then further annotated using the COG, GO and KEGG databases. COG results described that all the DEGs were ascribed to four functional categories: metabolism (1086 genes); information storage and processing (596 genes); cellular processes and signaling (719 genes); and poorly categorized genes (1484 genes). The greatest number of annotated genes were ascribed to metabolism, with replication recombination and repair (245 genes), inorganic ion transport and metabolism (209 genes), cell wall/ membrane/ envelope biogenesis (203 genes), carbohydrate transport and metabolism (196 genes), amino acid transport and metabolism (192 genes) and transcription (189 genes) being the largest (Fig. 3). GO enrichment grouped DEGs into three functional categories: biological process, many of which were involved in metabolic and cellular processes with only a few being involved in locomotion; cellular component, in which genes in the cell part and membrane part sub-categories were the most abundant; and molecular function, which included many genes associated with catalytic activity and binding as well as several associated with transporter activity, transcription regulator activity, structural molecule activity and antioxidant activity (Fig. 4). The subsequent KEGG analysis indicated that the greatest number of genes were enriched to metabolic pathway both in up-regulated DEGs (357 genes) and down-regulated DEGs (255 genes), among which carbohydrate metabolism and amino acid metabolism were enriched the greatest number of genes (Fig. 5).

### Assessment of candidate genes related to the VBNC state of Xanthomonas campestris pv. campestris

**Fig. 6:**
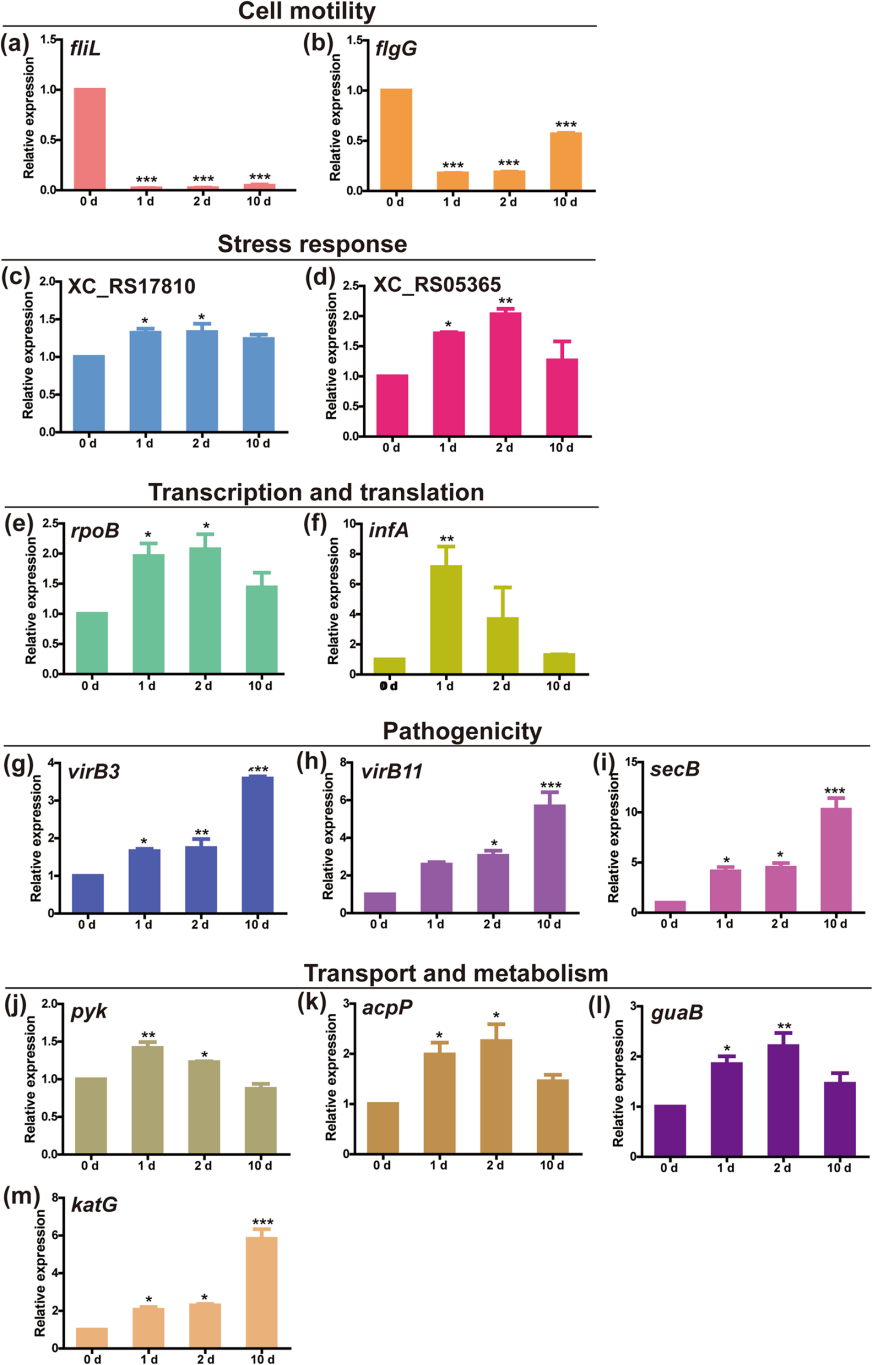
Assessment of gene expression by qPCR of 13 genes selected from RNA-seq results. The bar charts of (**a**) to (**m**) indicate the gene relative expression of *fliL*, *flgG*, XC_RS17810, XC_RS05365, *rpoB*, *infA*, *virB3*, *virB11*, *secB*, *pyk*, *acpP*, *guaB* and *katG*, respectively

Candidate DEGs possibly related to VBNC state of *Xanthomonas campestris* pv. *campestris* were identified according to the fold change and gene annotation analysis (Table 2). These genes were classified into cell motility (*fliL*, *flgB* and *flgG*), stress response (*clpP*, *clpXP*, *clpX*, XC_RS17810, XC_05365 and *rpfG*), transcription and translation (*infA*, *infB*, *infC*, *rpoB*, *dksA* and *greA*), pathogenicity (*virB3*, *virB11* and *secB*), and transport and metabolism (*pyk*, *acpP*, *guaB*, *katG*, XC_RS01110, XC_RS20570 and XC_RS18380), and these 5 functional categories could represent a broad range of biological processes. Thirteen of these genes were selected for further verification using qPCR. The genes regarding motility (*fliL* and *flgG*), flagellum related genes, were significantly down-regulated from 1 to 10 d, and the qPCR results were consistent with RNA-seq data (Fig. 6a, b and Table 2). Beyond cell motility-related genes, genes in the other categories selected were up-regulated overall (Table 2). The genes related to stress response (XC_RS17810 and XC_RS05365) and transcription and translation (*infA* and *rpoB*) were significantly up-regulated expression at early and developing stages of VBNC state (1 d and 2 d), but no significant changes were observed at the stable VBNC state (10 d) compared with that at 0 d (Fig. 6c, d, e and f). For the category of transport and metabolism, some genes (e. g. *pyk*, *acpP*, and *guaB*) were firstly up-regulated and then no changed during the 10-day induction period, and some genes (e. g. *katG*) were always up-regulated expression (Fig. 6j, k, l and m). Pathogenicity was of greatest concern for plant pathogenic bacteria, and the results showed that the pathogenicity relevant genes were up-regulated from 1 to 10 d (Fig. 6g, h and i). In general, the results of qPCR were consistent with those of transcriptome data (Fig. 6 and Table 2).

**Table 2:** Candidate genes likely to be associated with the VBNC state in *Xanthomonas campestris* pv. *campestris* based on RNA-Seq analysis

Classification	Qeury name	Gene name	Log_2_Fold change			Predicted function
			C1/CK	C2/CK	C3/CK	
Cell motility	XC_RS11405	*fliL*	-0.75	-1.74	-1.74	flagellar basal body-associated FliL family protein
	XC_RS11245	*flgB*	-0.57	-1.39	-1.31	flagellar basal body rod protein FlgB
	XC_RS11270	*flgG*	-0.90	-1.09	-1.049	flagellar basal-body rod protein FlgG
Stress response	XC_RS16490	*clpP*	2.42	2.18	1.63	ATP-dependent Clp endopeptidase proteolytic subunit ClpP
	XC_RS08995	*clpXP*	2.32	1.88	1.48	ClpXP protease specificity-enhancing factor
	XC_RS16485	*clpX*	1.85	1.42	0.67	ATP-dependent Clp protease ATP-binding subunit ClpX
	XC_RS17810	- ^a^	1.79	1.18	0.73	type II toxin-antitoxin system CcdA family antitoxin
	XC_RS05365	- ^a^	1.65	1.33	0.94	type II toxin-antitoxin system RelE/ParE family toxin
	XC_RS11730	*rpfG*	1.45	1.23	0.68	two-component system response regulator
Transcription and translation	XC_RS11160	*infA*	2.32	1.35	0.29	translation initiation factor IF-1
	XC_RS08025	*infB*	2.09	1.53	0.98	translation initiation factor IF-2
	XC_RS08265	*infC*	2.32	1.97	0.57	translation initiation factor IF-3
	XC_RS16935	*rpoB*	2.17	1.65	1.13	DNA-directed RNA polymerase subunit beta
	XC_RS09330	*dksA*	1.59	1.44	0.72	RNA polymerase-binding protein DksA
	XC_RS11780	*greA*	1.88	1.42	0.84	transcription elongation factor GreA
Pathogenicity	XC_RS08195	*virB3*	2.60	2.38	2.41	VirB3 family type IV secretion system protein
	XC_RS08180	*virB11*	2.17	1.80	1.16	P-type DNA transfer ATPase VirB11
	XC_RS01060	*secB*	1.94	1.89	1.51	protein-export chaperone SecB
Transport and metabolism	XC_RS04900	*pyk*	1.45	1.15	0.62	pyruvate kinase
	XC_RS15295	*katG*	1.23	1.10	1.14	catalase/peroxidase
	XC_RS16265	*acpP*	3.40	2.39	1.35	acyl carrier protein
	XC_RS09690	*guaB*	1.78	1.43	0.89	IMP dehydrogenase
	XC_RS01110	- ^a^	1.67	1.51	0.41	Fic family protein
	XC_RS20570	- ^a^	1.74	1.63	1.30	ABC transporter ATP-binding protein
	XC_RS18380	- ^a^	2.31	2.03	1.58	ABC transporter permease

CK indicates time zero, while C1-C3 indicate three time points after exposure to copper each associated with a different stage in the establishment of the VBNC state, namely early (1 d) and developing (2 d) stage induction, and the stable VBNC state (10 d), respectively

^a^ indicates no name has been allocated to these genes in the *Xanthomonas campestris* pv. *campestris* 8004 genome annotation database

## Discussion

Our previous results showed that *Xanthomonas campestris* pv. *campestris* could be induced into VBNC state by 50 µM CuSO_4_ and VBNC cell counts gradually reduced from 1 to 10 d [17]. Based on the above research results, transcriptome sequencing was performed at different time points of VBNC state (0 d, 1 d, 2 d, and 10 d) in this study. The heat map of similarity between samples showed that the sample of C2 and C3 was with high similarity and CK was the least similar to C3 and C2. Here, CK indicated time zero, while C1-C3 indicated three time points after exposure to copper each associated with a different stage in the establishment of the VBNC state, namely early (1 d), developing (2 d), and stable VBNC state (10 d), respectively. These results were consistent with previous results that the proportion of viable bacteria decreased from 0 to 10 d under copper treatment, and the physiological characteristics of bacteria gradually changed. In addition, Fig. 2 also showed that there were a large number of differentially expressed genes (DEGs) at different times.

The results of COG, GO, and KEGG indicated that most of DEGs were related to metabolism (Figs. 3, 4 and 5), which were consistent with the previous reports in *P. syringae* pv. *syringae* and *E. coli* [20, 25]. Among metabolism pathways, the most enriched pathways were inorganic ion transport and metabolism, and this was probably because copper was the inducer (Fig. 3). Moreover, the other most enriched metabolic pathways were related to carbohydrate transport and metabolism and amino acid transport and metabolism. Replication recombination and repair and transcription were mainly included in information storage and processing category, and cell wall/membrane/envelope biogenesis was the most abundant in cellular processes and signaling category. These meant that bacteria might need to synthesize important living substances, so that they could strive to survive. In metabolism-related pathways of KEGG pathway, there were significantly more up-regulated genes than down-regulated genes, indicating that the bacteria in VBNC state could maintain certain metabolic activity, which was also one of the characteristics of VBNC state [4, 5]. There were significantly more down-regulated genes of cell motility in cellular processes and signal transduction in environmental information processing than up-regulated genes of that, indicating that VBNC state was a kind of dormant state with no motility [29].

Among the DEGs, some genes that possibly were ascribed to VBNC state were screened (Table 2), and a total of 13 genes in different categories were verified by qPCR (Fig. 6). *fliL*, *flgB* and *flgG* were the flagellar genes and flagella are the major bacterial organelles for motility. The results showed that these genes were significantly down-regulated (Fig. 6a and b). It was speculated that bacteria kept themselves inactive to save energy, which was also a response of resistance to adversity. Intriguingly, pathogenicity relevant genes were significantly up-regulated (Fig. 6g, h and i). *virB3* and *virB11* were the essential virulence genes of type IV secretion system in *Agrobacterium tumefaciens* [30, 31] and *secB*, cytoplasmic chaperone, could be utilized by type I, II and IV secretion systems [32, 33]. Previous study also indicated that *E. coli* VBNC cells could potentially display pathogenicity [19]. VBNC bacteria could not be detected by conventional agar-plating methods, so undetected VBNC bacteria could pose a risk of serious harm to agricultural production. The type III secretion system effectors were the most important pathogenic factors for Xcc [34], and these genes showed no significant expressed in this study (data not shown). Thus, the pathogenicity of Xcc VBNC cells were still need to be determined, as well as the function of *virB3*, *virB11*, and *secB* in Xcc.

It could be speculated that stress response, transcription and translation, and transport and metabolism related genes were more likely to be associated with VBNC state. The variation trend of gene expression was consistent with the survival state of bacteria. Previously, the type II toxin-antitoxin (TA) system has been reported to play a role to induce the dormancy and persistence [29, 35]. As reported, two genes belonging to type II TA system, XC_RS17810 and XC_RS05365, were up-regulated expression in this study (Fig. 6c and d). Similarly, genes related to Clp family (*clpP*, *clpXP* and *clpX*) were also significantly up-regulated expression (Table 2), which were consistent with previous study that ClpP was accumulated in VBNC cells of *Legionella pneumophila* and the deletion of the C-terminal of ClpX resulted in reduced VBNC cell counts in *Salmonella enterica* [36, 37]. It has been reported that Lon/Clp could control the activity of TA modules [29], thus it was speculated that Clp family might also regulate TA module in Xcc, thereby triggered the VBNC state. For transport and metabolism, ABC transporter-related genes (XC_RS20570 and XC_RS18380) were up-regulated (Table 2) and ABC transporters were important for many different aspects of the bacterial physiology including import of essential nutrients and export of toxic molecules [38]. It was reported that *katG* were down-regulated and the authors explained that down-regulation expression of *katG* prevented the synthesis of sufficient catalase to eliminate the hydrogen peroxide produced during the sterilization of the media, thus not forming the colonies on the solid media and finally entering into VBNC state [39]. However, *katG* was up-regulated in this study (Fig. 6m) and we speculated that *katG* could reduce the hydrogen peroxide produced by intracellular metabolism to maintain VBNC state under adverse conditions. Phosphoenolpyruvic acid and ADP could produce pyruvic acid by pyruvate kinase to provide raw materials for the tricarboxylic acid cycle and *acpP* was involved in fatty acid synthesis [40]. Moreover, *pyk* and *acpP* were significantly up-regulated (Fig. 6j and k) and it was speculated that these genes related to metabolism might play an important role in maintaining VBNC state. The GuaB is IMP dehydrogenase, the essential enzyme to synthesize GTP, providing raw materials and energy for transcription and translation. Consistently, *guaB* and the genes related transcription and translation (*infA*, *infB*, *infC*, *rpoB*, *dksA* and *greA*) were significantly up-regulated in the VBNC state (Fig. 6l, e and f) to maintain the viability of VBNC cells, as well as to prepare for resuscitation after the removal of stress factors.

**Fig. 7:**
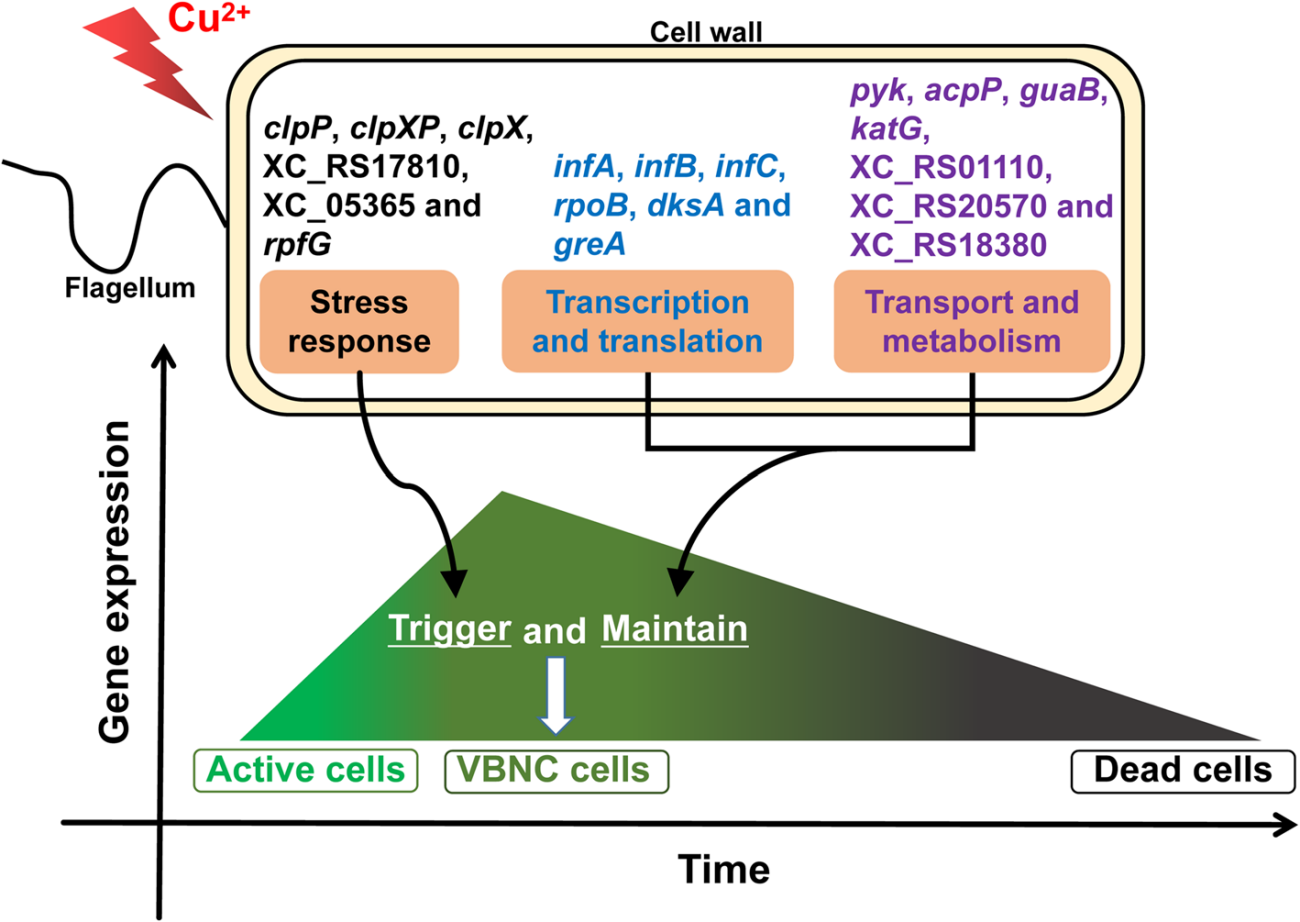
Dominant categories of differentially expressed genes in triggering and maintaining VBNC state identified from RNA-seq analyses in *Xanthomonas campestris* pv. *campeatris*. The x axis indicates induction time. With the induction of copper ions, the bacteria gradually enter into the VBNC state and eventually dead. The y axis indicates the gene expression level, and the genes that probably trigger and maintain VBNC state are up-regulated expression first, followed by stabilization in comparison of time zero. The genes related to stress response (*clpP*, *clpXP*, *clpX*, XC_RS17810, XC_05365 and *rpfG*) trigger the active cells to VBNC cells, while the genes involved in transcription and translation category (*infA*, *infB*, *infC*, *rpoB*, *dksA* and *greA*), as well as transport and metabolism category (*pyk*, *acpP*, *guaB*, *katG*, XC_RS01110, XC_RS20570 and XC_RS18380), play an important role in maintaining the VBNC state

The pathways probably associated with VBNC state were summarized in this study. It indicated that the genes related to stress response (*clpP*, *clpXP*, *clpX*, XC_RS17810, XC_05365 and *rpfG*) could trigger the active cells to VBNC cells, while the genes involved in transcription and translation category (*infA*, *infB*, *infC*, *rpoB*, *dksA* and *greA*), as well as transport and metabolism category (*pyk*, *acpP*, *guaB*, *katG*, XC_RS01110, XC_RS20570 and XC_RS18380), were ascribed to maintaining the VBNC state (Fig. 7). This is the first study to propose a model of VBNC state mechanism, which summarized not only the related pathways that might trigger and maintain VBNC state, but also the expression profiling of genes in different survival state of bacteria under stress. It provided a novel kind of gene expression profile and new ideas for VBNC state mechanism.

## Conclusions

The transcriptional profiling of *Xanthomonas campestris* pv. *campestris* in viable but nonculturable state was analyzed by RNA-seq, and the genes related to triggering and maintaining the VBNC state were validated by qPCR. This is the first study to propose a gene expression profiling to elucidate the mechanism of VBNC state, which summarized the related pathways that might trigger and maintain VBNC state. We believe that this study provides a novel and comprehensive gene expression profiling for VBNC state and lays the foundation for further exploring the mechanism of VBNC state to understand the bacterial survival strategy under stresses, as well as for disease management.

## Materials and methods

### Bacterial strains and culture conditions

*Xanthomonas campestris* pv. *campestris* (Xcc) strain 8004 [41] was routinely cultured in Luria–Bertani (LB) plate (5 g/L Yeast Extract, 5 g/L NaCl and 10 g/L Tryptone) at 28 °C for 48 h. For VBNC induction experiments, a single colony was picked into 10 mL LB broth and incubated at 28 °C with shaking for 12 h. Then, 2 mL of Xcc suspension was taken into 200 mL LB medium and cultured at the above conditions. Xcc cells were collected by centrifugation at 8000 rpm for 15 min, and washed with 0.85% (w/v) NaCl solution. The bacterial pellet was then resuspended with 0.85% (w/v) NaCl solution. Bacterial suspension was pipetted into 150 mL of 0.85% (w/v) NaCl solution supplemented with 50 μM copper sulphate (CuSO_4_) for OD_600_ = 0.18 and the resulting bacterial suspension were incubated at 28 °C without shaking in 250 mL flasks [17].

### RNA isolation and library construction

Xcc cells (OD_600_ = 2) were harvested by centrifugation at 12,000 rpm for 3 min at the time points of 0 d, 1 d, 2 d, and 10 d of CuSO_4_ treatment. Total RNA was extracted using the SV Total RNA Isolation System (Promega Corporation, Beijing, China) as the protocol of the manufacturer described and RNA stored immediately at -80 °C. The quantity and quality of RNA were determined by NanoDrop 2000 (Thermo Scientific, Beijing, China) and 1% agarose gels. The experiment was conducted using three separate biological replicates, with each treatment being represented by a pooled sample from three individual flasks.

The RNA library construction and sequencing itself was performed commercially by Shanghai Majorbio Bio-pharm Technology Co., Ltd (Shanghai, China). The ribosomal RNA was firstly removed using the Ribo-Zero Magnetic kit (Bacteria) from EpiCentre Biotechnologies (Illumina, CA, USA). The 5 μg of total RNA of each sample were used for strand-specific transcriptome libraries construction in conjunction with the TruSeq Stranded mRNA Sample Prep Kit (Illumina, CA, USA). Briefly, RNA was initially fragmented using fragmentation buffer before the first and second strand cDNA synthesis was conducted according to the kit protocol. When the second strand was synthesized, dUTP was incorporated in place of dTTP to generate the double stranded cDNA [42]. The Double-stranded cDNA structure was viscous end. So, End Repair Mix was added to generate the blunt end, and then a base was added to the 3’ end to connect the Y-shaped joint. Before PCR amplification, the second strand of cDNA was digested with UNG enzyme so that the library contained only the first strand of cDNA. The resulting libraries were then enriched by amplification of 15 cycles and quantified using Picogreen (Invitrogen, OR, USA) with the TBS380 Fluorometer (Invitrogen, OR, USA). The clusters were generated with the TruSeq PE Cluster Kit v3-cBot-HS (Illumina, CA, USA). Libraries were quantified and quality controlled by Bioanalyser 2100 (Agilent technologies, Beijing, China), and then subjected to paired-end sequencing on Illumina Hiseq 2500 platform with a target read length of 101 nucleotides.

### Data processing and transcriptome analysis

The raw sequencing reads were subjected to stringent quality filtering before mapping. The raw reads were first trimmed and quality controlled by SeqPrep software (https://github.com/jstjohn/SeqPrep) and Sickle (https://github.com/najoshi/sickle) with default parameters, which removed adaptor contaminants, reads with greater than 10% of ambiguous sequences represented by “N”, and reads with a quality score of less than 20. The resulting high-quality reads were then aligned to the *Xanthomonas campestris* pv. *campestris* 8004 genome database (NC_007086.1, NCBI) using Bowtie2 software (http://bowtie-bio.sourceforge.net/index.shtmL) based on Burrows-Wheeler algorithm with the criteria that reads should be uniquely matched to the genome allowing up to 2 mismatches without insertions or deletions [43].

Homology searches of the resulting sequences were performed with major public databases, including NCBI NT (nucleotide) database, NR (non-redundant) database, COG (Cluster of Orthologous Groups) database (http://www.ncbi.nlm.nih.gov), GO (Gene Ontology) database (http://amigo.geneontology.org/amigo), KEGG (Kyoto Encyclopedia of Genes and Genomes) database (http://www.genome.jp/kegg/) [44], and the Search Tool for the Retrieval of Interacting Genes (http://string-db.org/) to ascribe functional annotation. The GO terms were initially determined using the Blast2GO tool (http://www.blast2go.org) with the default parameters, while the enrichment analysis of GO terms was conducted using Goatools software (http://github.com/tanghaibao/GOatools) with the cutoff for the corrected p value (p_fdr) (no more than 0.05). The enrichment of KEGG pathways analysis was conducted using KOBAS software (http://kobas.cbi.pku.edu.cn/home.do) with a threshold of *P* < 0.05 [45].

The expression of genes was normalized as fragments per kilobase of transcript per million fragments mapped (FPKM) values using RSEM software (http://deweylab.biostat.wisc.edu/rsem) [46]. Compared to the transcriptional profiles obtained from the sample at time zero (CK), the genes with significantly altered expression were selected based on a false discovery rate (FDR) of less than 0.05 and the absolute Log_2_FC (fold change) value of no less than 1 using EdgeR (http://www.bioconductor.org/packages/2.12/bioc/htmL/edgeR.htmL) [47]. The differentially expressed genes common to different time points (1 d, 2 d, and 10 d) termed C1-C3 were then visualized in Venn Diagrams produced using the gplots R package (https://cran.r-project.org/web/packages/gplots/index.html).

### cDNA synthesis and quantitative real-time PCR (qPCR) analysis

The cDNA synthesis and qPCR analysis were performed according to a protocol from a previous study [17]. In brief, reverse transcription was performed using 2 μL of total RNA in conjunction with the PrimeScript RT Reagent Kit with gDNA Eraser (Takara, Beijing, China). The resulting cDNA was stored at -20ºC until required. The qPCR was conducted using an Applied Biosystems 7500 Fast Real-Time PCR System (Life Technologies, USA) with SYBR® Premix DimerEraser™ (TaKaRa, Japan). Each reaction volume (20 μL) contained 2 μL of diluted cDNA, 10 μL of SYBR Premix DimerEraser, 1 μL of each primer, 0.4 μL of Rox Reference Dye II, and 5.6 μL of sterile distilled water. The program of the qPCR was 95 °C for 30 s, followed by 40 cycles at 95 °C for 5 s, 56 °C for 30 s, and 72 °C for 30 s. The melt curve was generated with a series of fourfold cDNA dilutions. The PCR efficiency value (E) was calculated using a linear regression model where E (%) = (10^–1/slope^—1) × 100% [48], and the qPCR results normalized using the reference genes including *pbpA* and *ugpC* as detailed in a previous study [17]. The 2^−ΔΔCt^ method was used to calculate the relative expression level of each target gene [49]. When assessing the expression of candidate genes associated with the VBNC state, relative gene expression from samples at different VBNC stages were compared with the expression at 0 d. Each treatment was represented by three biological replicates, and the entire experiment conducted three times. The Primer Premier 5.0 software was used for design of the specific primers for qPCR according to the sequences retrieved from NCBI database. All primers used for qPCR analysis have been listed in Table S1.

## Supplementary Information

**Additional file 1: Table S1. **Primers used for the qPCR analysis conducted in the current study.

## Data Availability

The RNA-seq raw data of this study has been deposited in the Sequence Read Archive (SRA) database under accession number PRJNA797910 (https://www.ncbi.nlm.nih.gov/bioproject/PRJNA797910).
