# Supplementary material for: Transcriptional profiling of Xanthomonas campestris pv. campestris in viable but nonculturable state
Source: BMC Genomics. 2023 Mar 9;24:105. doi: 10.1186/s12864-023-09200-z (PMC9999588; doi:10.1186/s12864-023-09200-z)
Supplement: Supplementary file 1 — Additional file 1: Table S1. Primers used for the qPCR analysis conducted in the current study. [file 12864_2023_9200_MOESM1_ESM.docx]

**Table S1 Primers used for the qPCR analysis conducted in the current study**

| **Primer** | **Target Gene** | **Sequence (5’→3’)** | **Final Conc. (nM)** | **Product size (bp)** | **R^2^** | **Efficiency (%)** |
| --- | --- | --- | --- | --- | --- | --- |
| pbpART-F | *pbpA* | GCAACAACGGCGTGCTCAAC | 500 | 199 | 0.9995 | 95 |
| pbpART-R |  | CCTGGGCTATACCGAAGACA |  |  |  |  |
| ugpCRT-F | *ugpC* | AAGGTGTCCGCAAGGTCTACGA | 500 | 159 | 0.9983 | 103 |
| ugpCRT-R |  | TGCCCGCACTGATGTCCTCC |  |  |  |  |
| fliLRT-F | *fliL* | CGAAGAAACCCGAAAAGACCGAA | 500 | 95 | 0.9994 | 101 |
| fliLRT-R |  | CGCCAGCACAACGACACCGA |  |  |  |  |
| flgGRT-F | *flgG* | GCAGCAACGTCAATACGGTGGAAGA | 500 | 84 | 0.9958 | 102 |
| flgGRT-R |  | TGGAGATGGCCTTGGCGTTCATT |  |  |  |  |
| 3520RT-F | XC_RS17810 | CCGCTGAACCTGACCATCAACTCG | 500 | 134 | 0.9973 | 99 |
| 3520RT-R |  | ATCGCTCTGGCGCTGTGGCTGT |  |  |  |  |
| 1066RT-F | XC_RS05365 | TTTAGTCGCTTATCTGCTGGAACACG | 500 | 106 | 0.9963 | 103 |
| 1066RT-R |  | GCGCCCCATCAGTGGATTGTC |  |  |  |  |
| guaBRT-F | *guaB* | CGAGCCGTTTACGGTGAGCCC | 500 | 108 | 0.9995 | 96 |
| guaBRT-R |  | CCAACCAGTTCGCTGCCATCC |  |  |  |  |
| infART-F | *infA* | CACCACGTTCCGGGTCAAGC | 500 | 89 | 0.9996 | 92 |
| infART-F |  | TGAGGATGCGGATGTAGTTCTTGC |  |  |  |  |
| rpoBRT-F | *rpoB* | GGCGACCAGACGCTTGGATTC | 500 | 117 | 0.9969 | 95 |
| rpoBRT-R |  | GGCCGGATGAAGTTCAACCACC |  |  |  |  |
| virB3RT-F | *virB3* | ATGATCTTCAGGCTGCTTGGG | 500 | 107 | 0.9595 | 104 |
| virB3RT-R |  | TGCTTTCGATATTCGTTGGGACT |  |  |  |  |
| virB11RT-F | *virB11* | TTTACAAGAAGAATGTAGTGGTGGCC | 500 | 85 | 0.9993 | 106 |
| virB11RT-R |  | TTCGGAATGTGGTTGACAAGAGC |  |  |  |  |
| secBRT-R | *secB* | CTGCTGGCCCCGCTTTCACC | 500 | 100 | 0.9992 | 107 |
| secBRT-F |  | TCGGGCTGGTTGGCGTCATT |  |  |  |  |
| pykRT-F | *pyk* | TTGTCCGACCGCAAGGGCCTCA | 500 | 131 | 0.9947 | 107 |
| pykRT-R |  | CGGCAGAACGACACCGCGATGAA |  |  |  |  |
| katGRT-F | *katG* | GCAGTCGCACAATGGCGTCTTCAC | 500 | 127 | 0.9983 | 106 |
| katGRT-R |  | GCGGCCTTCGTAGGTTTCCTTGGTCT |  |  |  |  |
| acpPRT-F | *acpP* | TTCTCAGCTTCTTCGTCCGGGATT | 500 | 103 | 0.9953 | 100 |
| acpPRT-R |  | CGTTCGTCGATGACCTGGGTG |  |  |  |  |
